# Variability in the Beneficial Effects of Phenolic Compounds: A Review

**DOI:** 10.3390/nu14091925

**Published:** 2022-05-04

**Authors:** Itziar Eseberri, Jenifer Trepiana, Asier Léniz, Iker Gómez-García, Helen Carr-Ugarte, Marcela González, María P. Portillo

**Affiliations:** 1 Nutrition and Obesity Group, Department of Pharmacy and Food Sciences, University of the Basque Country (UPV/EHU) and Lucio Lascaray Research Institute, 01006 Vitoria-Gasteiz, Spain; itziar.eseberri@ehu.eus (I.E.); ikergomezgarcia98@gmail.com (I.G.-G.); helencarrugarte@gmail.com (H.C.-U.); mariapuy.portillo@ehu.eus (M.P.P.); 2 BIOARABA Health Research Institute, 01006 Vitoria-Gasteiz, Spain; asier.leniz@gmail.com; 3 CIBERobn Physiopathology of Obesity and Nutrition, Institute of Health Carlos III, 01006 Vitoria, Spain; 4 School of Nursing of Vitoria-Gasteiz, Osakidetza Basque Health Service, University of the Basque Country (UPV/EHU), 01006 Vitoria-Gasteiz, Spain; 5 Nutrition and Food Science Department, Faculty of Biochemistry and Biological Sciences, National University of Litoral and National Scientific and Technical Research Council (CONICET), Santa Fe 3000, Argentina; maidagon@fbcb.unl.edu.ar

**Keywords:** phenolic compounds, bioaccesibility, bioavailability, metabotype, chronobiology

## Abstract

When analysing the beneficial effects of phenolic compounds, several factors that exert a clear influence should be taken into account. The content of phenolic compounds in foods is highly variable, directly affecting individual dietary intake. Once ingested, these compounds have a greater or lesser bioaccessibility, defined as the amount available for absorption in the intestine after digestion, and a certain bioavailability, defined as the proportion of the molecule that is available after digestion, absorption and metabolism. Among the external factors that modify the content of phenolic compounds in food are the variety, the cultivation technique and the climate. Regarding functional foods, it is important to take into account the role of the selected food matrix, such as dairy matrices, liquid or solid matrices. It is also essential to consider the interactions between phenolic compounds as well as the interplay that occurs between these and several other components of the diet (macro- and micronutrients) at absorption, metabolism and mechanism of action levels. Furthermore, there is a great inter-individual variability in terms of phase II metabolism of these compounds, composition of the microbiota, and metabolic state or metabotype to which the subject belongs. All these factors introduce variability in the responses observed after ingestion of foods or nutraceuticals containing phenolic compounds.

## 1. Introduction

The beneficial effects of phenolic compounds on health have been demonstrated in epidemiological and preclinical studies [1,2,3,4]. However, when considering the positive impact of phenolic compounds in humans, high inter-individual variations in the biological responses to dietary phenolic compound intake and supplementation have been commonly reported [5]. This fact hampers the translation of current knowledge about these compounds into dietary advice and hinders health claims for the general population. Consequently, more research on the factors that underlie these differences is needed.

Phenolic content in foods is very variable, which directly affects their individual dietary intake [6,7]. Furthermore, following oral intake, the in vivo effects of phenolic compounds depend, in part, on their bioaccessibility, which is defined as the amount that is available for absorption in the gut after digestion [8]. They also rest on their bioavailability, defined as the proportion of the molecule that is available after digestion, absorption and metabolism [9].

Among the external factors that alter phenolic content in foodstuffs, we find plant origin, cultivation technique, climate and type of food matrix. Chemical interactions between phenolic compounds and other components of the diet can have significant consequences. Moreover, there is great inter-individual variability in the response to phenolic compound intake due to personal differences in metabolism (i.e., genetic variants of enzymes involved in phase II metabolism), microbiota composition, the individual metabolic status, or the metabotype to which the subject belongs, among other factors [10,11,12].

**Figure 1:**
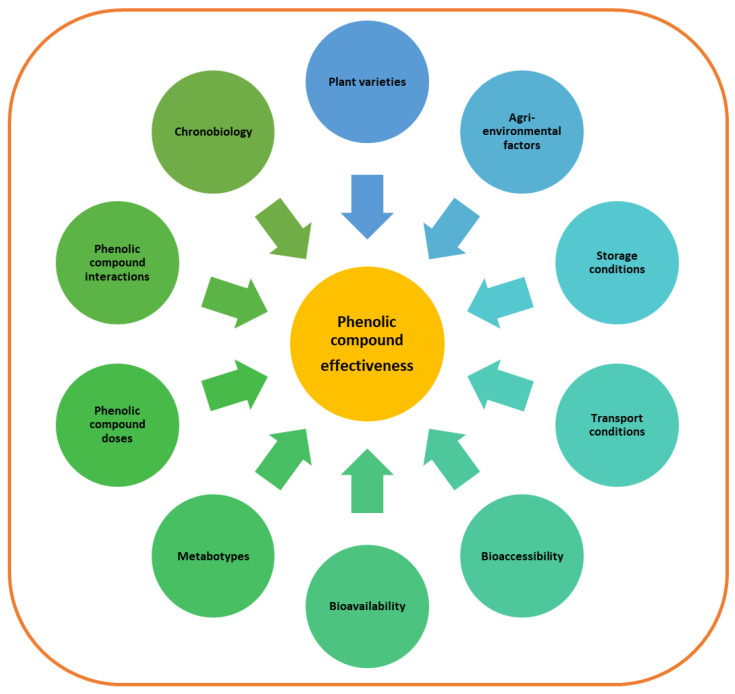
Factors affecting phenolic compound effectiveness.

All these elements cause an array of responses after the ingestion of foods or nutraceuticals containing phenolic compounds. Consequently, it is very difficult to offer precise recommendations about their consumption. The present review does not intend to carry out an exhaustive reexamination of the reported papers that address the role of all these factors on the effects of the main phenolic compounds ingested by humans, because it is not possible to reflect all this information in a single paper. For this reason, we have defined two aims: First, to provide a general overview of the difficulties found in standardising the recommendations for phenolic compound intake, by revising the factors that play a key role in the effects of these molecules (Figure 1). Second, to provide scientific evidence to support this issue by describing several reported studies.

## 2. Chemistry of Phenolic Compounds

Phenolic compounds constitute a group of substances that are widely present in the plant kingdom, where more than 8000 are known, with different chemical structures and activities [13,14]. They can be found in vegetables, seeds, fruits, nuts, red wine, tea and many other food sources. Structurally, phenolic compounds are secondary plant metabolites characterised by at least one aromatic ring with one or more hydroxyl groups attached [15]. There are two basic pathways involved in the biosynthesis of phenolic compounds, the shikimic acid pathway and the malonic acid pathway. In plants, the main pathway is the former. The shikimate pathway consists of seven reaction steps, beginning with an aldol-type condensation of phosphoenolpyruvic acid (PEP) from the glycolytic pathway, and D-erythrose-4-phosphate from the pentose phosphate cycle, to produce 3-deoxy-D-arabino-heptulosonic acid 7-phosphate (DAHP). A key branch-point compound is chorismic acid, the final product of the shikimate pathway. They are generally produced as defence mechanisms against pathogens, protection from excess ultraviolet radiation and as attractants for pollinators. Complex phenolic compounds are also important structural components of plants [16].

Phenolic compounds with more than one phenolic group are called polyphenols. Generally, phenolic compounds are found in conjugated form with one or more sugar moieties, as glycosides, linked through OH group (*O*-glycosides) or through carbon–carbon bonds (*C*-glycosides). The sugar bonds could be monosaccharides, disaccharides or even oligosaccharides, being the most common glucose, although it could be bound to galactose, rhamnose, arabinose, xylose or glucuronic acid [17]. 

**Figure 2:**
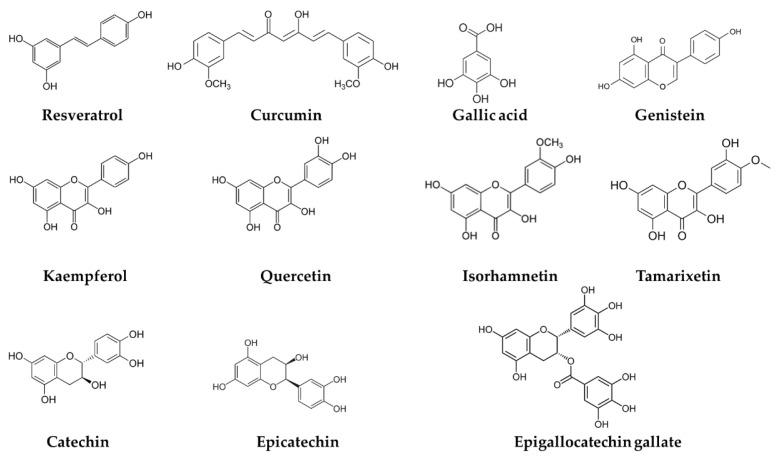
Phenolic compounds showing different chemical structures.

Based on the structure of the aglycones, they are first divided into flavonoids and non-flavonoids. Flavonoids are 15 carbon compounds configured as C_6_-C_3_-C_6_, generally as two aromatic rings, connected by three carbons, and they are subdivided into several groups (flavonols, flavones, isoflavones, flavanones, flavan-3-ols and anthocyanidins) [18]. Non-flavonoids are composed of one or two aromatic rings and are classified as phenolic acids, which contain a C_6_-C_1_ carbon skeleton, hydroxycinnamates with a structure of C_6_-C_3_, hydrolysable tannins with one or two aromatic rings and stilbenes, with a more complex structure of C_6_-C_2_-C_6_ [14,19]. Some phenolic compounds with different chemical structures are shown in Figure 2.

**Figure 3:**
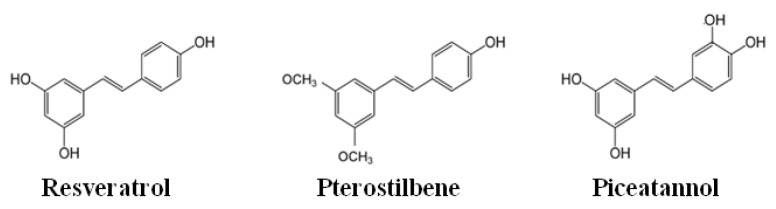
Chemical structures of resveratrol, pterostilbene and piceatannol.

The chemical structure of phenolic compounds can influence their bioavailability and biological actions. A good example is the slight differences in the chemical structure of resveratrol, piceatannol and pterostilbene. Although they are chemical analogues, piceatannol and pterostilbene have been shown to have greater activity [19,20], probably due to their higher resistance to intestinal and hepatic metabolism, given by the differences in the amount of hydroxyl and methoxyl groups among them [21] (Figure 3). In addition, Rice-Evans and co-workers (1996) hypothesised that the different antioxidant activity of phenolic compounds could be related to their ability to act as radical scavengers in relation to their chemical structures [22]. 

## 3. Factors That Affect Phenolic Content and Composition of Foods

**Figure 4:**
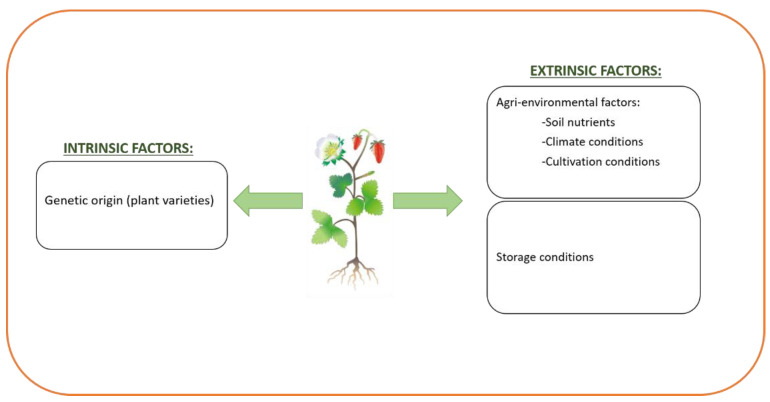
Main factors that affect phenolic content and composition of foods.

There are numerous factors that have a clear influence on the amount and composition of phenolic compounds present in plants (Figure 4). Among them, there are factors intrinsic to the plant itself (genetic origin) that lead to interspecies differences, and varieties of the same product. For instance, in the case of the lettuce varieties, whereas romanine, baby and iceberg have a poor content of antioxidant phenolic substances (flavanols and derivatives of caffeic acid), leaf oak and *lollo rosso* show high contents [23].

There are also factors extrinsic to the plant, linked to the growing circumstances (agri-environmental factors) and to the post-harvest storage conditions. Regarding growing conditions, the presence or absence of certain nutrients in the soil can affect the phytochemical composition of fruits and vegetables, both qualitatively and quantitatively. As an example, it is known that the content of calcium in soil induces phenolic metabolism and anthocyanin accumulation in grapes [24]. Boron availability also affects the phenolic content of plants substantially. In fact, this mineral can increase the key enzyme phenylalanine ammonia-lyase (PAL), which augments the anthocyanin biosynthesis [25].

Climate is another key aspect, and as a result, fruits from the same variety cultivated in different areas present different contents of phenolic compounds. If we take grapes as an example, it has been demonstrated that high temperatures during the growing stages can decrease anthocyanin synthesis [26]. Conversely, the water status at the flowering stage of the grape cycle has a positive effect on phenolic compound synthesis, whereas the synthesis of anthocyanins and phenolic compounds increases with water deficits during maturity stages [27].

The influence of seasonal variations has been studied in peach. Rahmati et al. (2014) analysed the changes in peach components (carbohydrates, organic acids and phenolic compounds) when exposed to long-term drought in semi-arid climate conditions during the spring and summer of 2011 in Golmakan (Iran) [28]. The authors reported that phenolic compound concentration (mainly anthocyanin and chlorogenic acid) increased under severe drought. When they studied the effect of this severe stress treatment, they observed that compared to low-stress treatment, the phenolic compound concentrations increased about 62–85%.

The effect of climate on phenolic compound composition in berries has also been studied. González-Domínguez et al. (2020) analysed the chemical profile (sugars, organic acids, phenolic compounds and mineral elements) of five different varieties of strawberries that were cultivated in two consecutive campaigns under different climatic conditions [29]. They reported that the content of anthocyanins and the total amount of phenolic compounds were greater under higher rainfall and more extreme temperatures. Ferreira et al. (2020) performed a study to evaluate the composition of three Portuguese cultivars of elderberry over the course of three years [30]. They observed that the harvesting year had a stronger influence on the phenolic composition and antioxidant activity than the cultivar one. They suggested that the climatic conditions, especially the water status, hardly modified the chemical composition of the elderberries. Regarding sunlight exposure, the greatest content of total flavonoids and anthocyanins was found in fruit juices subjected to southern exposure, followed by northern exposure fruits [31].

With regard to cultivation conditions, in the study previously described, and published by González-Domínguez et al. (2020), the authors compared the composition of strawberries grown in two soilless systems: a closed system with recirculation of the nutrient solution and an open system without this recirculation [29]. After determining the phenolic content in the fruits, they only found slight differences in the concentration of several anthocyanins and phenolic acids. Along this line, “Golden Delicious” apples from organic cultivars showed a greater phenolic content than those from conventional cultivars [32]. It is important to mention a study by Mulero et al. (2010), where the authors claimed that significant differences were observed between unripe organic and nonorganic grape cultivars, though these differences disappeared when grapes reached the ripening stage [33]. Thus, although the aforementioned studies have shown that this agricultural practice could increase the amount of phenols in the cultivar, Winter et al. (2006) did not find significant differences between organic and conventional cultivars [34]. Considering that this is an important factor affecting phenolic composition, further research is required.

The maturity stage of the different fruits and vegetables also exerts a significant influence on the phytochemical composition. Although a general pattern for all products has not been found, in general terms, immature fruits show a lower level of phenolic compounds. In a study carried out with four blackberries cultivars from the germplasm bank of the National Institute of Agricultural Research (INIAP), Quito (Ecuador), the total content of polyphenols decreased during the maturation process of the four blackberries cultivars. Nevertheless, not all the polyphenols followed the same pattern. Thus, whereas the content of flavonoids decreased with the ripening process, the content of anthocyanins increased [35]. In another study carried out with *Cucurbita moschata* Duchesne pumpkin harvested at different ripening stages (young, mature, ripened) in Algeria, the authors reported that the amount of phenolic acids was dependent on the maturity stage. Therefore, according to the results, caffeic acid, cinnamic acid, coumaric acid and dihydroferulic acid increased from young to mature fruits. Nevertheless, a significant decrease in the levels of these molecules occurred at the end of the ripeness [36]. In the same line, coumaric acid content significantly increased from green to mature stages in the juice of three different pomegranate cultivars of Turkey [37].

In addition, changes in the phenolic compound composition can also occur during the processing and conservation of foods. Conservation is generally carried out at low temperatures, which induces the expression of enzymes responsible for the biosynthesis of some phenolic compounds [38]. For this reason, the content of some constituents increases occasionally during the conservation of certain fruits and vegetables. On the other hand, post-harvest treatments with ozone or irradiations with UV light or gamma radiation entail an increase in the biosynthesis of phytochemicals in most cases. Thus, irradiation with UV light induces the accumulation of resveratrol in grapes [39]. Oracz et al. (2015) reported that technological processes applied to cocoa beans, including fermentation, drying and roasting, affect the final content of phenolic compounds, leading to their decomposition [40].

As a consequence of the variability of the phenolic compound profiles found in fruits and vegetables, important differences in phenolic compound intakes can be observed among subjects, even when following a similar dietary pattern, and thus in the beneficial effects on health derived from this intake. This makes it difficult to offer precise recommendations about the most advisable foodstuffs aimed at reaching specific amounts of phenolic compounds. The great variability poses an important limitation in terms of comparing the effects of phenolic compound extracts and reducing the effects of these extracts.

## 4. Factors Affecting Phenolic Compound Bioaccessibility

Most phenolic compounds are glycosylated, and the attached sugar moiety is usually released before absorption. The amount of phenolic compounds available for absorption after ingestion can be affected by several factors, such as the presence of other compounds in the diet, such as fibre, lipids, proteins and digestible carbohydrates (Table 1). Soluble dietary fibre can prolong gastric emptying time, and thus delay the absorption of phenolic compounds in the small intestine. Dietary fibre may also reduce the rate of the molecules absorbed, by physically trapping the phenolic compound within the fibre matrix due to the interaction between polar groups from the phenolic compounds and the fibre polysaccharides [41]. Along this line, Tew et al. (1996) observed that a high wheat-fibre diet produced lowered plasma genistein (55%) 24 h after having taken a single soy-rich food, presumably due to increased viscosity and hydrophobic interactions between them [42]. Moreover, Manach et al. (2005) reported that phenolic compound absorption differed among adults after the same intake of aglycone equivalents as a plant extract or as a whole food [9].

With regard to the dietary lipids, it should be pointed out that although the majority of the phenolic compounds are water-soluble, the apolar ones, such as curcumin, resveratrol, xanthones and some flavonoid aglycones, are micellarised with dietary fat. Accordingly, Guo et al. (2013) reported on the bioaccessibility of quercetin, which presents lipophilic properties, that it is increased at 45% in subjects who consumed the aglycon supplement combined with a fat-rich (15 g) breakfast, due to enhanced phenolic compound micellization and absorption in the intestine [43]. Lesser et al. (2004) showed increased (57%) quercetin bioaccessibility in pigs fed with a diet that provided 17% fat, compared with pigs that received a diet that provided 3% fat [44]. In the case of resveratrol, contradictory results have been reported. Vaz-da-Silva et al. (2008) carried out a study where subjects, who followed either a high-fat content meal or eight-hour fasting, were treated with *trans*-resveratrol [45]. They concluded that, although a large inter-individual variability in the *trans*-resveratrol pharmacokinetic parameters was observed, the amount of the phenolic compound absorbed was similar under both feeding conditions. Conversely, La Porte et al. (2010) studied the pharmacokinetics of *trans*-resveratrol (2000 mg twice daily), administered with a standard or a high-fat breakfast to eight healthy subjects, and reported that the high-fat breakfast significantly decreased the *trans*-resveratrol absorption when compared with the standard breakfast [46].

Regarding dietary proteins, it seems that those phenolic compounds that contain a high number of hydroxyl groups display a strong affinity for these molecules. It has been suggested that such interactions consist of the formation of hydrogen bonds and hydrophobic relations between hydroxyl groups of the phenolic compounds and the carbonyl groups of the proteins [47]. The protein-phenolic compound complex formed might reduce the absorption of the latter [48]. However, Lang et al. (2021) in a study carried out in rats, reported that blueberry anthocyanin absorption could increase 1.5–10 times when administered intragastrically with α-casein [49]. Moreover, other authors reported that protein-rich foods do not have an impact on phenolic compound bioaccessibility. Consequently, it is clear that this remains a controversial issue.

In other studies, the authors used beverages rich in proteins instead of isolated proteins. Along this line, Draijer et al. (2016) carried out a study where 35 healthy males received a grape extract incorporated into a dairy drink, soy drink (both containing 3.4% proteins) or protein-free drink. The authors reported that the intake of phenolic compounds in combination with hyperproteic drinks had no effect on the bioavailability of epicatechin, gallic acid, isorhamnetin or resveratrol [50]. Keogh et al. (2007) studied the effect of milk proteins on the bioavailability of cocoa phenolic compounds in humans. For this purpose, 24 subjects consumed chocolate phenolic compounds with or without milk, and the authors reported that proteins did not modify the average concentration of catechins and epicatechins [51]. In the same line, it was reported that the addition of milk had no significant effect on black tea catechin bioaccessibility [52]. By contrast, Serafini et al. (2009) observed that the absorption of blueberry phenolic compounds (caffeic and ferulic acid) was reduced when they were ingested with milk [53]. An important limitation of these studies is that, although the observed effect could be the result of an interaction between proteins and phenolic compounds, the involvement of other milk components such as lipids or carbohydrates cannot be discarded.

Phenolic compound absorption can also be modified by dietary carbohydrates. Schramm et al. (2003) demonstrated in humans that dietary carbohydrates increased the absorption of cacao flavanols by 40%, whereas protein or lipid-rich meals did not induce this effect, perhaps because the glycoside uptake might be enhanced by the sugars in the diet [54]. In this study, the authors suggested that the increased absorption of phenolic compounds, which were consumed after a carbohydrate-rich meal, could be due to the influence of carbohydrates on gastrointestinal motility and/or enzyme secretion.

**Table 1:** Effects of the diet macromolecules on the phenolic compound bioccessibility.

Food Compound	Type of Interaction	Effect	References
Dietary fibre	Prolongation of gastric emptying (soluble fibres)	↑ absorption time	[42]
	Increase in viscosity (soluble fibres)	↓ % absorption	[9]
	Physical trapping		
Lipids	Micellization of polar phenolic compounds	↑ % absorption	[43,44]
		= % absorption	[45]
		↓ % absorption	[46]
Proteins	Protein-phenolic compound complex formation	↓ % absorption	[53]
		↑ % absorption	[49]
		= % absorption	[50,51,52]
Digestible carbohydrates	Absorption facilitation of phenolic compound glycosides by sugars	↑ % absorption	[54]

Altogether, these results show the great influence of the dietary pattern on the bioaccessibility of phenolic compounds. As a consequence, a similar intake of these compounds can lead to important differences in blood concentrations, and thus in biological effects, depending on the composition of the diet, or the fact that phenolic compounds, present in a nutraceutical product, are ingested in meals or out of them.

## 5. Factors Affecting Phenolic Compound Bioavailability

**Figure 5:**
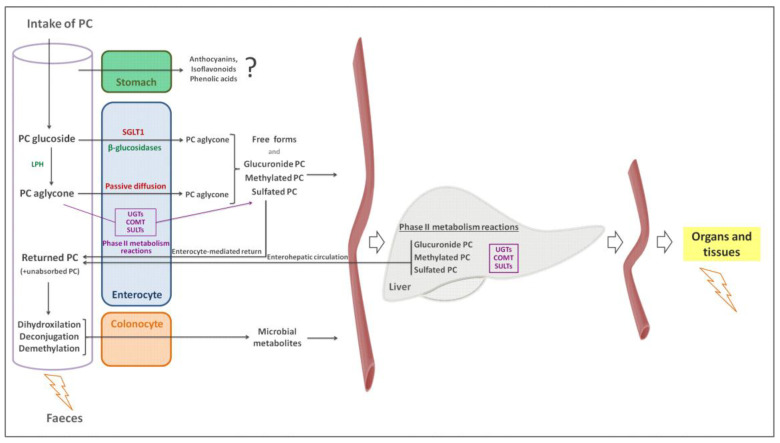
Schematic representation of phenolic compound metabolism. PC: phenolic compound; LPH: lactase-phlorizin hydrolase; SGLT1: sodium-dependent glucose transporter 1; UGTs: uridine 5-diphosphate glucuronosyltransferases; COMT: methylation by catechol-O-methyltransferase; SULTs: sulphotransferases.

In addition to the factors that affect phenolic compound bioaccesibility, and consequently bioavailability, there are other elements described in this section that affect the latter. The vast majority of phenolic compounds are rapidly and extensively metabolised after their absorption (Figure 5) [14]. In general, phenolic compounds appear in foodstuffs in glycosylated forms, and they are hydrolysed to the aglycone form in the small intestine by two mechanisms [55,56]. In the first one, lactase-phlorizin hydrolase (LPH) deglycosylates phenolic groups, releasing free aglycones which are ready to be absorbed by enterocytes. In the second mechanism, glycosides are carried by the sodium-dependent glucose transporter 1 (SGLT1) (Figure 5) to be further cleavaged by cytosolic β-glucosidases [57]. Due to their fast appearance in plasma, it seems that a part of some phenolic compounds such as anthocyanins, isoflavonoids and phenolic acids, are absorbed in the stomach, although intestinal absorption is considered the main one.

As previously mentioned, phenolic compounds undergo rapid metabolism in both enterocytes and the liver. The cytochrome P450 (CYP) family of enzymes catalyse phase-I reactions, which include oxidation, reduction and hydrolysis [58]. Additionally, they undergo extensive phase-II detoxification reactions, which include glucuronidation by uridine 5-diphosphate glucuronosyltransferases (UGTs), methylation by catechol-O-methyltransferase (COMT) and sulphuration by cytosolic sulphotransferases (SULTs). These phase II metabolic reactions serve the organism to reduce their potential toxic effect and to facilitate their biliary and urinary excretion by increasing their hydrophilicity [59]. Non absorbed phenolic compounds reach the colon, where they are subjected to extensive microbial metabolism [14,60].

As a result of metabolism, the amounts of metabolites in plasma and tissues are often higher than those of the parent compounds [61]. Although, in general, phase II metabolism is considered an important limitation for the use of phenolic compounds as therapeutic tools, it is currently known that some metabolites can be active and responsive in part, to the effect of the parent compound [62,63,64,65,66,67,68,69,70].

Taking into account that not all metabolites can act as active compounds [64,68,70,71,72], inter-individual differences in the production of phase II metabolites and microbial metabolites are of great importance to assess the effectiveness of phenolic compounds. Regarding this issue, variations in the expression of genes coding for the enzymes involved in phase II metabolism have been described in animals and in humans suffering some diseases. Liu et al. (2012) observed that the plasma area under the curve (AUC) of mangiferin was significantly higher in diabetic rats than in the control group after a single oral dose of 400 mg/kg [73]. The authors measured the gene expression of the main phase II enzymes in the liver, and they observed that the expression of *Ugt1a3*, *Ugt1a8*, *Ugt2b8* and *Sult1a1* was higher in diabetic rats. By contrast, *Comt*, *Ugt2b6*, *Ugt2b12* and *Sult1c1* mRNA levels were lower.

Dostalek et al. (2011) provided evidence that diabetes in humans significantly reduced the mRNA expression, protein level and activity of hepatic UGT2B7, suggesting fewer glucuronidation reactions in the liver of diabetic patients [74]. Moreover, Yalcin et al. (2013) studied hepatic sulphotransferase expression and activity in healthy subjects and in individuals diagnosed with steatosis, diabetes, diabetic cirrhosis or alcoholic cirrhosis [75]. They found that the capacity of SULT1A1 diminished significantly in livers with steatosis and in more seriously diseased liver tissue. This reduction in SULT1A1 activity could result in reduced concentrations of sulphated metabolites and increased concentrations of the parent compound. In addition, regarding the isoform SULT2A1, its activity was found to be significantly decreased only in the group showing cirrhosis induced by alcohol, but not in steatotic or diabetic cirrhotic livers when compared with non-fatty controls.

With reference to microbial metabolites, the information about their activity is scarcer. Our group has demonstrated that, at physiological concentrations, dihydro-resveratrol is as effective as the parent compound in preventing triglyceride accumulation in hepatocytes [69]. Microbial metabolite production can vary depending on the composition of gut microbiota, that in turn is modified by a great number of factors such as sex, age, ethnicity, diet, physical activity, stress, drugs for disease treatment and so on. The inter-individual differences in metabolites produced by gut microbiota are a very interesting issue. In this field of research, special attention should be paid to “metabotypes”. These are metabolic phenotypes identified by the presence of specific metabolites derived from the catabolism of phenolic compounds by particular gut microbiota, in terms of composition and functionality. According to this definition, two metabotypes which have been unequivocally identified have been described: a) equol producers vs. equol non-producers in the metabolism of isoflavones and b) producers of only Uro-A (UM-A) vs. producers of Uro-A, isourolithin-A (IsoUro-A) and vs. urolithin-B (Uro-B) (UM-B) and vs. urolithin non-producers (UM-0) in the metabolism of ellagic acid [76]. Although the existence of metabotypes for other phenolic compounds has been proposed by several authors, their existence has not been adequately demonstrated.

Concerning the metabolism of isoflavones, it has been reported that, as opposed to non-producers, individuals who produce equol or *O*-desmethylangolensin as metabolites resulting from daidzein metabolism show the beneficial effects on cardiometabolic markers attributed to daidzein, one of the main phenolic compounds in soy [77]. Taking into account that the percentage of equol producers has been estimated to be around 30% in Caucasians, and 50–60% in the Asian population, this metabotype can partly explain differences in the effectiveness of daidzein described in several studies [5].

With regard to the metabolism of ellagitannins, González-Sarrias et al. (2017) reported that, whereas the chronic consumption of an ellagitannin-rich pomegranate extract did not induce beneficial effects on blood lipids in a cohort of adult overweight-obese males and females when individuals were distributed in different urolithin metabotypes, the subjects with UM-B metabotype displayed a significant hypolipidemic effect, as opposed to those showing UM-A or UM-0 metabotypes [78]. The same group published a further study demonstrating, for the first time, that individuals’ differential capacity to metabolise ellagic acid derivatives into urolithins depends mainly on age. In fact, aging leads to a progressive reduction in UM-A, a metabotype concomitant with an increase in UM-B up to 30–40 years of age, after which the UM distribution remains constant [79].

In conclusion, differences in the activity of enzymes involved in phase II metabolism, as well as in microbiota composition and functionality can lead to discrepancies in the ratio of parent compound/derived metabolites found in plasma and tissues, which in turn can have important consequences in terms of phenolic compound effectiveness. Moreover, the metabotype to which each individual belongs determines the presence or absence of some specific metabolites required to observe the beneficial effects of the parent compound. Consequently, this is one of the reasons that can help explain inter-individual variability in the response to phenolic compound intake. Unfortunately, there is not enough knowledge concerning the presence of alternative metabotypes for other phenolic compounds, as well as for the distribution of the metabotypes according to sex, age and ethnicity, to name but a few. Moreover, it should be pointed out that future studies aimed at identifying new metabotypes ought to be performed in very large cohorts in order to avoid misleading results, which represents an important limitation for researchers [79].

## 6. Dosage Considerations

One of the most important and hard aspects of the study of the potential biological effects of phenolic compounds is to establish the best dosage that bears no risk of producing toxic consequences. Far from the traditional belief that the higher the dose, the greater the biological effect, we currently know that this is not always true and that the relationship between the dose and the effectiveness is more complex than previously thought. Regarding this issue, several studies have reported unexpected results.

In a study carried out by our group, resveratrol was administered at 6, 30 or 60 mg/kg body weight/day to rats fed with a hypercaloric diet, in order to analyse its potential anti-obesity effect [80]. Whereas the lowest dose did not exert any effect, the dose of 30 mg/kg body weight/day significantly reduced the size of subcutaneous, epididymal, perirenal and mesenteric adipose depots. Thus, in this range, a dose-response pattern was observed. However, the effect of the highest dose (60 mg/kg body weight/day) did not induce a greater consequence than the intermediate dose (30 mg/kg body weight/day). These results show that, in some cases, a “plateau” is reached in a specific range of doses.

In other cases, an increase in the dose of the phenolic compound leads to a reduction in the magnitude of the effect. Thus, Cho et al. (2012) addressed a study devoted to analysing the anti-obesity and anti-steatotic effects of resveratrol, where mice were fed with a high-fat diet supplemented or not with this phenolic compound (0.02% or 0.005%) [81]. The authors observed that the supplementation of resveratrol at the lowest dose significantly suppressed body weight gain after three weeks, whereas the highest dose was surprisingly not effective. With regard to the effect on adipose tissue, perirenal and mesenteric depots were significantly reduced in mice supplemented with either the high dose or the low dose of resveratrol, although only the low dose was able to effectively reduce the weight of the epididymal and retroperitoneal depots. Similarly, the low dose of resveratrol appeared to be more effective in reducing the number and size of liver fat droplets than the highest one. These results show that, in a specific range of doses, lower doses of resveratrol have more beneficial effects on adiposity and hepatic steatosis compared to higher ones.

Finally, it is important to mention that a significant number of studies have shown that low and high doses of phenolic compounds have opposite effects. This is based on the phenomenon known as hormesis, which is defined as a biphasic response to phytochemicals in relation to physiological functions [82,83]. Hormesis has been clearly demonstrated with respect to the anti-oxidant effect of phenolic compounds. Dudley et al. (2009) reported that at doses of 2.5 or 5 mg/kg body weight/day, resveratrol protected against heart ischaemia by inducing upregulation of anti-apoptotic and redox proteins, whereas at higher doses (>25 mg/kg body weight/day), it caused the downregulation of redox proteins and the upregulation of pro-apoptotic proteins [84]. Furthermore, it has been shown that high doses of resveratrol cause atherosclerotic lesions whilst lower measures have protective effects in hypercholesterolaemic rabbits [85]. Additionally, curcumin has been described as a powerful antioxidant agent in tumour cells at doses below 5 µg/mL, but its effect, becomes harmful at high doses since it increases oxidative stress by reactive oxygen species (ROS) generation [86]. Hormesis has also been observed in other phenolic compound-derived health effects. In a study performed by Oh et al., (2006) the authors analysed whether kaempferol could be useful against oestrogen-related diseases (i.e., breast cancer, osteoporosis and cardiovascular diseases) [87]. After testing the effect of this compound in oestrogen-sensitive human breast cancer cells, the authors were able to identify a biphasic response. Finally, El Touny et al., (2009) observed in their study that genistein exerted a biphasic regulation of prostate cancer growth and metastasis, suggesting that hormone-dependent cancers could be especially sensitive to the hormetic effects of phenolic compounds [88].

Another important aspect that should be analysed regarding the relationship between the amount of phenolic compounds received by the subjects and the derived effects is the administration pattern, mainly in the case of nutraceuticals and functional foods enriched with these compounds. In this context, Chow et al. (2003) found that initially, there was no difference in the maximum plasma concentration of epigallocatechin gallate after a unique dosage of 800 mg or a double dosage of 400 mg twice a day [89]. However, after the repeated four-week treatment period, the area under the curve of free epigallocatechin gallate in participants treated with the unique dose was 60% higher than that of the subjects treated with the fractioned dosage. The authors hypothesised that non-enzymatic degradation, microbial metabolism, methylation reactions and intestinal efflux of the compound could be implicated in this observation, although this fact should be further studied. Furthermore, in a study aimed at analysing the pharmacokinetic of *Ginkgo biloba* extract in rats, the authors observed that a repeated administration of 600 mg/kg of the extract for eight days led to a significant increase in plasma concentration of its main phenolic compounds (quercetin, kaempferol and isorhamnetin/tamarixetin) when comparing to a one-day administration of the same amount [90]. Along the same line, Ferruzzi and colleagues (2009) observed that a repeated administration of grape seed phenolic compound extract increased the plasma concentration of gallic acid, catechin and epicatechin around two-fold in rats [91]. In fact, they showed that the maximum concentration of the three phenolic compounds and their main metabolites increased in a dose-dependent manner. These data suggest that a repeated administration of phenolic compounds leads to a higher maximum plasma value of the parent compound.

The reasons underlying all the facts described above are not very clear. One of the most probable explanations is related to the rapid metabolism suffered by phenolic compounds after their intake. A saturation of phenolic compound metabolism enzymes after a high dose administration could be responsible for a modification in the rate of parent compound/derived metabolites found either in plasma or tissues. Taking this into account, and as described in several studies, some phenolic compound-derived metabolites show biological activity, where the modification in the ratio of parent compound/derived metabolites can lead to differences in the effects exerted by phenolic compound intake.

The vast majority of the reported studies that address phenolic compound supplementation have been carried out with a single dose of phenolic compound. Consequently, further studies testing more than one dose are required in order to establish thresholds of exposure and the best range which may characterise beneficial and adverse effects. Similarly, the adequate posology for each phenolic compound ought to be defined. This information is crucial for functional food and nutraceutical producers.

## 7. Interactions among Phenolic Compounds and other Bioactive Molecules

Although a great number of the reported studies addressing the beneficial effects of phenolic compounds focus on the administration of an individual compound, it is important to bear in mind that, in fact, a combination of different phenolic compounds is present in foodstuffs and phenolic compound extracts. Moreover, even with the supplementation of a nutraceutical product that provides a single phenolic compound, this becomes mixed with others coming from the diet in the intestine. Consequently, the interactions among phenolic compounds must be taken into account when analysing the effects of these molecules. In fact, the absorption, metabolism and biological effects of each one may be modified.

With regard to absorption, a competitive mechanism among phenolic compounds can occur, and one molecule can reduce the absorption rate of the other. This phenomenon is supported by studies such as the one conducted by Silberberg et al. (2005), where the authors aimed to analyse the bioavailability of quercetin and catechin in rats fed diets supplemented with an equal dose (45 mg/d) of each of these polyphenols, administered alone or in association. The authors observed a competitive interaction between quercetin and catechin at the digestive level, leading to a reduction in the intestinal absorption of quercetin and a possible delaying of catechin absorption over time [92]. Along the same line, Orrego-Lagarón et al. (2016) reported a decrease in the absorption of naringenin and quercetin when both polyphenols were co-administrated by using an experimental design based on the cannulation of the intestine in mice and the perfusion of a solution containing naringenin, quercetin or both polyphenols together [93].

Concerning metabolism, it has been demonstrated that some phenolic compounds are able to inhibit the metabolism of others. Thus, de Santi et al. (2000) demonstrated that resveratrol sulphation was inhibited by the flavonoid quercetin, thus improving its bioavailability [94]. Moreover, glucuronidation can also be inhibited by quercetin, although to a lesser extent [95] (Figure 3). Consistent with these results, in a study carried out in rats by our research group, we found the reduction in adipose tissue size from different anatomical locations (subcutaneous, perirrenal, epididymal and mesenteric) induced by the combination of both compounds was significantly higher than the sum of the individual effects of each polyphenol on these tissues [96]. This synergistic effect was probably due to the reduction in sulphation metabolism of resveratrol induced by quercetin. Likewise, Mertens-Talcott and Percival observed that ellagic acid and quercetin displayed a synergistic effect with resveratrol in diminishing the progression of cancer in human leukemia cells (MOLT-4) [97]. The authors proposed that a combination of the three phenolic compounds, although not on an individual basis, could be responsible for the anti-carcinogenic effect observed with phenolic compound-rich foodstuffs.

**Figure 6:**
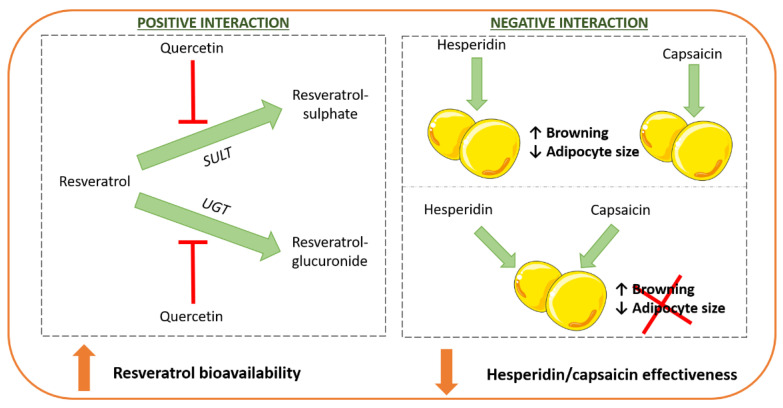
Schematic examples of interactions between phenolic compounds: The combination of phenolic compounds can increase/reduce the effectiveness of each compound when administered separately. SULT: sulphotransferases; UGT: uridine 5-diphosphate glucuronosyltransferases.

Along this line, Mosqueda-Solís et al. (2018) observed that whereas hesperidin and capsaicin, administered individually, reduced adipocyte size and induced white adipose tissue browning in rats fed with a western diet, the combination of both compounds decreased the effectiveness of each compound to exert these effects [98] (Figure 6). Concerning the mechanisms underlying this negative interaction, it can be hypothesised that hesperidin may override the effects of capsaicin through its transient receptor potential cation channel 1 (TRPV1). In fact, compared with the control rats, rodents treated with hesperidin displayed lower *Trpv1* gene expression levels in the inguinal adipose tissue. Nevertheless, the involvement of other mechanisms responsible for these interactions cannot be ruled out.

Phenolic compounds are also able to interact with other bioactives (conjugated fatty acids, amino acids, bioactive peptides, etc.). Sometimes these interactions lead to the loss of the biological effects. Thus, in previous studies conducted by our research group both in in vitro and in vivo experiments, it was observed the individual effects of resveratrol and conjugated linoleic acid on adipose tissue size and the activity of enzymes involved in adipose tissue triglyceride metabolism were abolished when these two active molecules were administered together [99,100,101]. The reasons that support this fact were not analysed. By contrast, in other cases, the interaction induces a synergistic effect. Bruckbauer et al. showed that the amino acid leucine acted synergistically with low concentrations of resveratrol to increase sirtuin-1 and AMPK activities, thus resulting in improved insulin sensitivity, and increased muscle glucose and palmitate uptake in vitro and in vivo [102]. Moreover, in a further study, the same group demonstrated that the synergistic effect between leucine and resveratrol was not unique but could also be extrapolated to other polyphenols with structural similarities. The single aromatic ring structure bound to a carboxylic group appeared to play a key structural role in this synergy [103].

According to these interactions, the effects of a phenolic compound can be expected to be different depending on the presence of other bioactive molecules in the diet. This fact justifies an inter-individual variation in the response to phenolic compounds contingent upon the dietary pattern. This knowledge is important in order to design the most appropriate combinations of bioactive molecules in the treatment or prevention of diseases.

## 8. Phenolic Compounds and Chronobiology

The circadian rhythm has emerged as a potential key modulator of phenolic compound bioactivity. At the same time, the intake of these compounds can modulate biological rhythms [104]. However, this is quite a new topic that still requires further research.

Escobar-Martínez et al. (2021) addressed a study aimed at evaluating whether the plasma bioavailability of phenolic compounds from a grape seed phenolic compound extract was affected by the administration time [105]. For this, they used healthy rats and a model of rats that exhibited metabolic syndrome induced by cafeteria diet feeding. The study showed a strong influence of the circadian rhythm on the plasma bioavailability of phase-II metabolites. More precisely, the rodents treated during their resting phase (light phase) displayed a higher average concentration of total phenolic compounds, compared with those treated during their active phase (dark phase). Similarly, the levels of phase II metabolites were higher during the day phase. Moreover, compared with sulphated and methylated metabolites, a higher concentration of glucuronide metabolites was observed, which could be attributed to a higher expression of UGTs at early light hours, compared with SULTs and COMTs. Furthermore, these results might indicate that the expression of SULTs and COMTs are not as sensitive to time administration as UGTs. These results are in good accordance with those reported by other authors. Zhang et al. (2009) observed a higher expression of phase II enzymes in mice during the light phase, and Zmrzljak and Rozman (2012) found that whereas mRNA levels of UGTs were maximal during the light time, those of SULTs were maximal during the light to dark transition [106,107].

According to these results, it can be stated that time can have a role in the effect of phenolic compounds on health since this factor can influence, together with others, the ratio of parent compound/metabolites. This knowledge is important in order to make recommendations about fruit and vegetable consumption, and also to determine the precise time of day a nutraceutical with phenolic compounds should be taken, as occurs with some drugs.

## 9. Concluding Remarks

The present review shows that the biological effects on health induced by phenolic compound intake, either as natural dietary components or as a supplementation using nutraceuticals or functional foods, depend on a great number of factors, such as the cultivar, the climate, the transport conditions, the phenolic compound interactions, the inter-individual variability and the circadian rhythm. It is evident that, although we know which these factors are, the relative importance of each one is unknown and that not enough research has been undertaken to clearly establish optimum conditions for intake that lead to the best effects on health.

Due to the fact that the amount of phenolic compounds naturally present in foods is very variable, and based on the varieties of the same foodstuff, the cultivar conditions, the soil composition, the climate and the storage and transport conditions, even when recommending a specific dietary pattern designed to reach a high intake of phenolic compounds, the actual consumption can be quite variable from one subject to another. This limitation is very difficult to avert. However, in the case of the phenolic compound supplements, either provided as nutraceuticals or used to elaborate functional foods, this variability in phenolic compound sources should be taken into account, and an extensive work on selection should be done in order to better settle on these sources and to adequately standardise phenolic compound extracts.

Further research is also needed to instruct in the administration of adequate doses, not only to find the best beneficial effect/administered dose ratio but also to avoid unexpected effects that bear negative consequences, as in the case of hormetic responses. Whereas the latter is not very relevant when phenolic compounds enter via the naturally phenolic compound-rich foodstuffs present in the diet, the fact that their intake usually does not reach the requisite levels to induce a hormetic response should be considered when phenolic compounds are supplemented. The vast majority of the reported studies has been carried out by using just one phenolic compound dose. Thus, in order to address this problem, new preclinical studies testing a wide range of doses are needed.

In addition, as in the case of some drugs, recommendations about the way to administer the phenolic compound supplements, in terms of the time of day, whether they are added or not to meals, as a single or repeated dose/day, together with guidance on the meal composition should be provided by nutraceutical producers to ensure the best response. Nevertheless, to date, not enough scientific evidence is available to set these standards, and thus, further research is warranted to compare the effects of phenolic compound supplements administered at different times of the day, in the meals or out of them, as well as to precisely analyse the influence of diet composition on the phenolic compound bioaccesibility and effectiveness. 

On the other hand, although the low bioavailability of phenolic compounds has been considered as a limitation for the use of these compounds as effective tools in the management of several diseases, it has been demonstrated that some metabolites derived from phenolic compound metabolism (phase II metabolism and microbiota metabolism) are active compounds that contribute, together with their parent compound, to the beneficial effects attributed to the latter. In this regard, further studies should be addressed to analyse the potential activities of phenolic compound metabolites because the available information is scarce so far, and the majority of the studies has been restricted to cancer research. 

As explained in this review, a great inter-individual variability has been found in the response to phenolic compounds from subject to subject, due to differences in the activity of phase II enzymes and microbiota composition. However, the existence of different metabotypes in the population has only been adequately established in the case of isoflavones and ellagitannis. Consequently, more research must be addressed to analyse whether metabotypes for other phenolic compounds also exist. Moreover, the potential influence of sex, age and ethnicity should be considered. This research is needed in order to use phenolic compounds in the frame of personalised nutrition, which is to optimise exclusive phenolic compound recommendations to best suit each subject, since not all the proposals may exert the same benefits in all individuals.
